# Early quantitative stress-perfusion cardiac magnetic resonance reveals perfusion abnormalities consistent with coronary microvascular dysfunction in MINOCA patients with otherwise normal cardiac magnetic resonance findings

**DOI:** 10.1038/s41598-026-67231-4

**Published:** 2026-08-31

**Authors:** Ramin Sahar, Joel Lenell, Bertil Lindahl, Anne-Marie Montelius, Johan Berglund, Karl-Henrik Grinnemo, Andrei Malinovschi, Tomasz Baron

**Affiliations:** 1 Department of Medical Sciences, Clinical Physiology, Uppsala University, Uppsala, 751 85 Sweden; 2 Department of Medical Sciences, Cardiology, Uppsala University, Uppsala, 751 85 Sweden; 3 Uppsala Clinical Research Center, Uppsala University, Uppsala, 751 85 Sweden; 4 Department of Surgical Sciences, Radiology, Uppsala University, Uppsala, 751 85 Sweden; 5 Department of Surgical Sciences, Molecular Imaging and Medical Physics, Uppsala University, Uppsala, 751 85 Sweden; 6 Department of Surgical Sciences, Cardiothoracic Surgery, Uppsala University, Uppsala, 751 85 Sweden

**Keywords:** Cardiac magnetic resonance (CMR), Quantitative stress-perfusion CMR (Q-CMR), Myocardial infarction with non-obstructive coronary arteries (MINOCA), Coronary microvascular dysfunction (CMD), Cardiology, Diseases, Medical research

## Abstract

Coronary microvascular dysfunction (CMD) is a proposed contributor to myocardial infarction with non-obstructive coronary arteries (MINOCA), but the value of early quantitative stress-perfusion CMR (Q-CMR) in this setting is not fully established. We aimed to determine the prevalence of quantitative perfusion abnormalities consistent with CMD using Q-CMR in patients with a working diagnosis of MINOCA and otherwise normal conventional CMR findings. Of 46 consecutive MINOCA patients referred for CMR between 2021 and 2024, 23 had normal findings on conventional CMR at rest and were included. They were compared with age- and sex-matched healthy controls without coronary artery disease, confirmed by coronary CT angiography. All participants underwent dual-sequence first-pass perfusion CMR at rest and during regadenoson stress. Fully automated quantitative perfusion analysis was used to quantify myocardial blood flow (MBF) and myocardial perfusion reserve (MPR). CMR at rest was considered normal in the absence of edema, late gadolinium enhancement, or other structural abnormalities. The MINOCA patients (mean age 63 ± 10 years, 61% female) were scanned a median of 15 days (IQR 10–37) after admission. Compared with controls, they had lower MPR (1.88 ± 0.59 vs. 2.30 ± 0.55; *p* = 0.013) and lower stress MBF (2.56 ± 0.81 vs. 2.96 ± 0.53 mL/g/min; *p* = 0.044), while rest MBF was similar. 43% of MINOCA patients had quantitative perfusion findings consistent with CMD according to previously proposed CMR thresholds, compared with none of the controls. In conclusion, early Q-CMR identifies reduced stress MBF and MPR in MINOCA patients with otherwise normal conventional CMR findings and reveals perfusion abnormalities consistent with CMD in nearly half of these patients, suggesting that microvascular dysfunction may contribute to myocardial injury in a subset of MINOCA patients.

## Introduction

Myocardial infarction with non-obstructive coronary arteries (MINOCA) represents a diagnostically challenging syndrome with multiple underlying mechanisms, including plaque disruption, coronary embolism, vasospasm, and coronary microvascular dysfunction (CMD). Accurate identification of the underlying mechanism is essential because prognosis, secondary prevention, and treatment strategies differ substantially according to the underlying etiology^1^. Standard cardiac magnetic resonance (CMR) is recommended to identify conditions such as myocarditis, Takotsubo syndrome, or cardiomyopathies, which account for a substantial proportion of MINOCA presentations^2–4^. However, a considerable subset of patients has normal conventional CMR findings despite fulfilling diagnostic criteria for myocardial infarction, leaving the underlying mechanism unresolved.

CMD has emerged as a plausible explanation for ischemic symptoms and troponin elevation in patients without obstructive epicardial coronary artery disease. Functional abnormalities in the coronary microcirculation - such as impaired vasodilatory capacity, increased resistance, and endothelial dysfunction - can cause reduced perfusion during stress, while resting flow remains preserved. Although increasingly recognized, CMD remains challenging to diagnose in clinical practice, partly because conventional imaging techniques lack the sensitivity to detect subtle microvascular abnormalities^5^.

Quantitative stress-perfusion CMR (Q-CMR) enables absolute measurement of myocardial blood flow (MBF) and myocardial perfusion reserve (MPR), providing a non-invasive assessment of myocardial perfusion and potential abnormalities of coronary microvascular function. Compared with conventional visual perfusion analysis, quantitative perfusion has demonstrated improved diagnostic accuracy, reproducibility, and observer independence^6^. Technical advances such as dual-sequence acquisition^7–8^, automated pixel-wise perfusion mapping, and fully automated post-processing workflows^1,5,9–11^ have markedly improved reproducibility and facilitated clinical implementation of Q-CMR^12^. Although quantitative stress-perfusion CMR has become an established tool for assessing coronary microvascular function, with validation against invasive physiological measurements^6^, its role in patients with MINOCA remains incompletely defined^9,12^. Previous studies have demonstrated impaired stress myocardial perfusion in patients with MINOCA, including persistent abnormalities detected years after the index event^13^. However, most investigations have evaluated heterogeneous MINOCA populations or patients at later stages after presentation^13–14^. Consequently, little is known about the prevalence of quantitative perfusion abnormalities in patients with otherwise normal conventional CMR findings during the early diagnostic phase following MINOCA.

We hypothesized that quantitative perfusion abnormalities consistent with CMD may be under-recognized among MINOCA patients with otherwise normal conventional CMR findings, and that quantitative stress-perfusion CMR would identify perfusion abnormalities consistent with impaired coronary microvascular function compared with healthy individuals. Therefore, we prospectively compared stress myocardial blood flow, rest myocardial blood flow, and myocardial perfusion reserve between MINOCA patients and age- and sex-matched healthy controls using automated quantitative dual-sequence Q-CMR. To our knowledge, this is the first study to evaluate early quantitative stress-perfusion CMR specifically in patients with MINOCA and completely normal conventional CMR findings, addressing an important diagnostic gap in patients in whom routine CMR does not identify an underlying cause of myocardial injury.

## Methods

### Study population

Consecutive patients with a working diagnosis of MINOCA referred for CMR at Uppsala University Hospital between January 2021 and August 2024 were included in the Swedish Web-system for enhancement and Development of Evidence-based care in Heart disease Evaluated According to Recommended Therapies (SWEDEHEART) registry^15^, which prospectively collects nationwide data from patients admitted to coronary care units or other specialized facilities because of suspected acute coronary syndrome. A working diagnosis of MINOCA was defined as: (1) fulfillment of the Universal Definition of Myocardial Infarction criteria, (2) absence of coronary artery stenosis ≥ 50%, and (3) no identified alternative cause for the MI presentation^2,4^.

The control group consisted of age- and sex-matched healthy individuals without angina and with normal coronary computed tomography (CT) angiography, defined as CAD-RADS 0 (no stenosis or plaque) according to the CAD-RADS™ 2.0^16^ consensus document, performed within 5 years prior to inclusion as a part of the Swedish CardioPulmonary bioImage Study (SCAPIS)^17^.

### Coronary angiography

All patients underwent invasive coronary angiography during hospitalization and prior to CMR as part of the diagnostic work-up. Angiographic data were retrieved from the SWEDEHEART registry.

### CMR protocol

The CMR imaging was performed on a Philips 1.5 T Multiva scanner with a 32-channel dStream Torso coil (Philips Diagnosis & Treatment, Best, The Netherlands).

Patients with a working diagnosis of MINOCA and healthy volunteers underwent first-pass perfusion CMR during pharmacological stress with intravenous regadenoson (standard dose of 400 µg) and at rest following intravenous theophylline to reverse vasodilatation. Perfusion imaging used a vendor-supplied research dual-sequence acquisition^5^, in which a single bolus of gadobutrol (Gadovist, Bayer AB, Solna, Sweden, 0.05 mmol/kg) was administered during the simultaneous acquisition of two distinct image series: one for arterial input function (AIF) measurement (voxel size 6.8 × 2.6 × 10.0 mm3, saturation time 30 ms, single basal slice) and another for myocardial enhancement (voxel size 2.6 × 2.6 × 10.0 mm3, saturation time 90 ms, three short-axis slices basal/midventricular/apical). ECG-triggered acquisition was performed at one frame per heartbeat during 80 heartbeats under free breathing. The single-shot spoiled gradient echo readouts were made with a flip angle (FA) 15°, and echo time (TE) 1.2 ms, a repetition time (TR) of 2.7 ms, and field of view (FOV) 288 × 316 mm^2^, bandwidth 865 Hz/pixel, partial Fourier factor 0.75, and compressed sensing under-sampling factor 2.3.

**Fig. 1:**
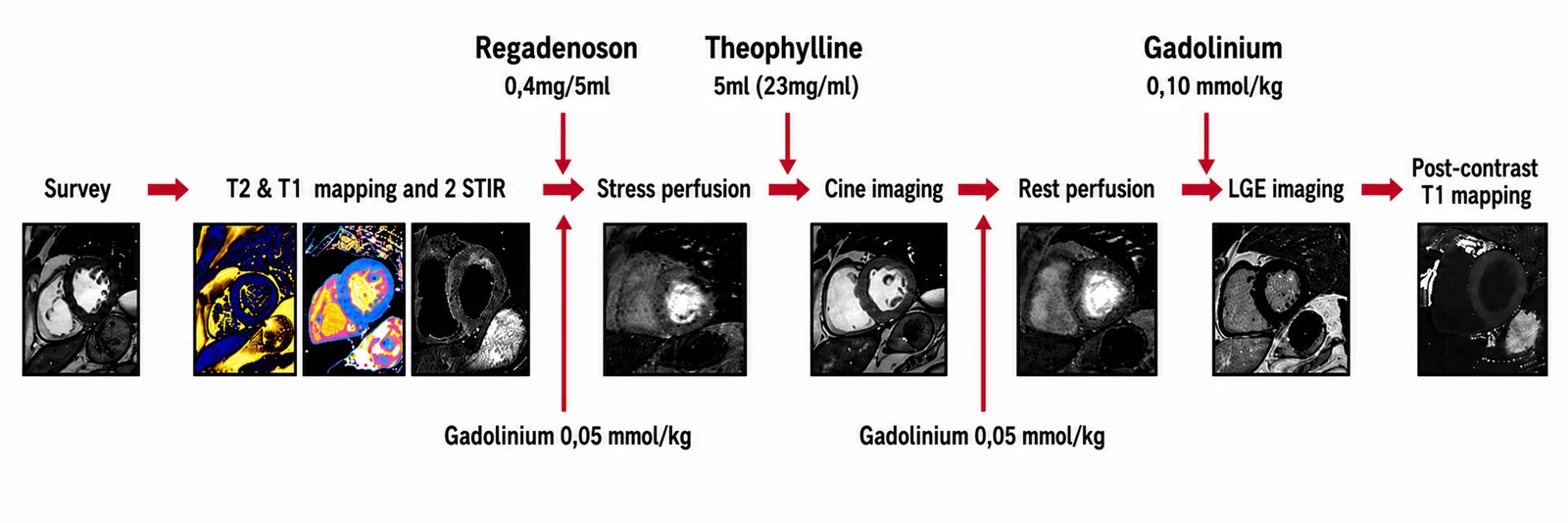
Regadenoson CMR stress-perfusion protocol. Survey imaging, native T1 mapping, native T2 mapping, and STIR sequences are acquired first, followed by stress perfusion imaging. Regadenoson is administered to induce hyperemia, and theophylline is subsequently given to reverse its effects. Cine imaging and rest perfusion are then performed, and late gadolinium enhancement is acquired last. The total duration of the protocol is approximately 50 min.

Additional routine sequences included Late Gadolinium (LGE), Phase-sensitive inversion recovery (PSIR), cine balanced steady-state free precession (cine bSSFP), short tau inversion recovery (STIR), T2 mapping and T1 mapping before and after contrast administration (see Fig. 1).

Patients were abstained from caffeine for 24 h and nicotine for 12 h prior to the CMR examination. Adequate hemodynamic response was defined as a systolic blood pressure decrease by > 10 mmHg and/or a heart rate increase by > 10 bpm.

Fully automated quantitative perfusion analysis was performed using commercially available Circle CVI42 software (version 5.13, Circle Cardiovascular Imaging Inc., Calgary, AB, Canada). The software uses automated image segmentation and pixel-wise perfusion mapping to quantify myocardial blood flow (MBF) during stress and rest conditions, as well as myocardial perfusion reserve (MPR), defined as the ratio of stress to rest MBF. Quantitative perfusion values were obtained from the dual-sequence first-pass perfusion images at the global, coronary territory, and segmental levels. Previous studies have validated automated quantitative perfusion mapping against invasive coronary physiology and demonstrated good diagnostic performance and reproducibility^6,18–19^. All automatically generated perfusion maps and myocardial contours were visually reviewed by the investigators before final analysis. Examinations with insufficient image quality for reliable quantitative perfusion analysis were excluded. The reproducibility of the quantitative perfusion analysis was evaluated separately as described in the Statistical methods. Perfusion findings consistent with coronary microvascular dysfunction were defined using previously proposed quantitative CMR thresholds of stress MBF < 2.25 mL/g/min or MPR < 2.0 in the absence of any other pathological CMR findings. These thresholds were derived from previous validation studies demonstrating agreement between quantitative CMR perfusion parameters and invasive coronary physiological measures^6^. Although originally validated in mixed populations with suspected coronary artery disease rather than specifically in MINOCA, they represent the most widely used non-invasive CMR criteria for identifying perfusion abnormalities consistent with impaired coronary microvascular function. A normal CMR examination was defined as the absence of myocardial edema, late gadolinium enhancement, or other pathological findings (e.g., cardiomyopathies).

### Ethics

This study was approved by the Regional Ethical Review Boards in Lund (Dnr 2021 − 01517) and Stockholm (Dnr 2020–05953; amendment Dnr 2024-06698-02). All participants provided written informed consent. The study followed the principles of the 1975 Declaration of Helsinki.

### Statistical methods

Continuous variables were assessed for normality using the Shapiro–Wilk test and visual inspection of histograms and Q–Q plots. Normally distributed variables are presented as mean ± standard deviation (SD), whereas non-normally distributed variables are reported as median with interquartile range (IQR). Categorical variables are summarized as counts and percentages.

Between-group comparisons of continuous variables (MINOCA vs. healthy controls) were performed using the independent-samples Student’s *t*-test for normally distributed data and the Mann–Whitney *U* test for non-normally distributed data. Categorical variables were compared using the Chi-square test or Fisher’s exact test when appropriate. Global myocardial blood flow (MBF), coronary territory–specific MBF, and myocardial perfusion reserve (MPR) were compared using the same criteria for test selection based on distribution.

To evaluate whether differences in quantitative perfusion parameters between groups remained independent of potential confounding factors, multivariable linear regression analyses were performed with global stress MBF and global MPR as dependent variables. Models were adjusted for age, sex, and smoking status, which differed between groups and were considered clinically relevant potential confounders. Multicollinearity was assessed using variance inflation factors (VIF). Standardized effect sizes were calculated for the primary global perfusion outcomes using Hedges’ g with 95% confidence intervals to quantify the magnitude and precision of between-group differences. Interobserver reproducibility of the automated quantitative perfusion analysis was evaluated in a subset of 20 participants (10 MINOCA patients and 10 healthy controls) using the intraclass correlation coefficient (ICC) derived from a two-way random-effects model with absolute agreement. Bland–Altman analyses were additionally performed for global stress MBF, global rest MBF, and global MPR to assess agreement between observers and identify potential systematic differences.

All tests were two-sided, and a *p*-value < 0.05 was considered statistically significant. Given the exploratory nature of the study, no formal sample size calculation or adjustment for multiple comparisons was performed; therefore, the findings should be interpreted as hypothesis-generating. Statistical analyses were performed using IBM SPSS Statistics version 28 (IBM Corp., Armonk, NY, USA).

## Results

**Fig. 2:**
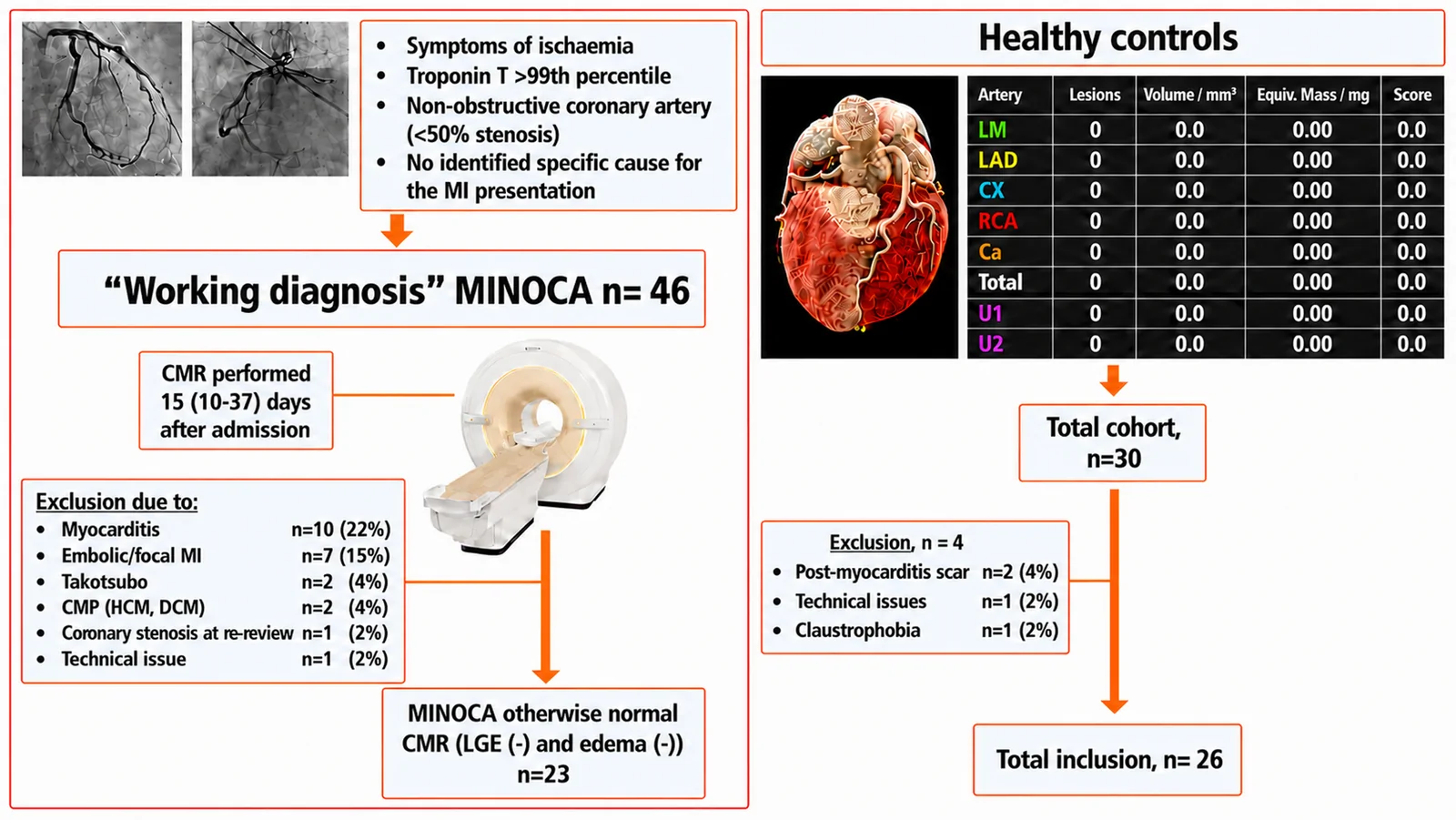
Flow chart illustrating the selection of the study population, including patients with a working diagnosis of MINOCA and healthy controls.

Stress-perfusion CMR was performed in 46 patients with a working diagnosis of MINOCA and 30 healthy age-matched controls. The selection process is illustrated in Fig. 2.

Among the MINOCA cohort, 10 patients (22%) were diagnosed with myocarditis, 7 (15%) with embolic MI, 2 (4%) with Takotsubo syndrome, and 2 (4%) with other conditions and were therefore excluded. One patient was excluded due to the presence of coronary stenosis on re-evaluation of the coronary angiography, and one due to suboptimal image quality. In the control group, two individuals with evidence of silent myocardial infarction, one with claustrophobia, and one with poor scan quality were excluded.

The final study population thus comprised 23 MINOCA patients with normal routine CMR findings and 26 healthy controls. The MINOCA patients underwent CMR a median of 15 days (IQR 10–37) after hospital admission. Mean age and sex distribution did not differ between MINOCA patients and healthy controls (62.6 ± 9.7 years, 61% females and 61.1 ± 3.6 years, 54% females, respectively). Compared with controls, MINOCA patients had higher rates of smoking, cardiovascular risk factors, medications, and higher concentrations of cardiac biomarkers (Table 1).

**Table 1:** Baseline characteristics of patients with MINOCA and otherwise normal CMR scan, and healthy controls.

Clinical data	MINOCA *n* = 23	Healthy controls *n* = 26	*p*-value
Demographics			
Age, y, mean± SD	62.6 ± 9.7	61.1 ± 3.6	0.492
Female sex, n (%)	14 (60.9)	14 (53.8)	0.620
BSA, m^2^, mean ± SD	1.93 ± 0.25	1.97 ± 0.25	0.542
Smoking, n (%)	10 (43.5)	1 (3.8)	< 0.001
Co-morbidities,* n (%)*			
Hypertension	10 (43.5)	4 (15.4)	0.030
Diabetes	2 (8.7)	0 (0)	0.125
Hyperlipidemia	10 (43.5)	3 (11.5)	0.011
Medications n (%)			
ACE-I/ARB	13 (56.5)	4 (15.4)	0.010
Beta-blockers	6 (26.1)	0 (0)	0.040
Calcium channel blockers	4 (17.4)	1 (3.8)	0.141
Statins	19 (82.6)	1 (3.8)	< 0.001
Laboratory results, median (IQR)			
Troponin T, ng/L	157 (71;265)	6 (5;9)	< 0.001
NT-proBNP, ng/L	203 (76;514)	76 (50;98)	0.006

BSA, body surface area; ACE-I, angiotensin converting enzyme inhibitor; ARB, angiotensin receptor blocker.

There were no significant differences in left ventricular volumes, mass, systolic function (LVEF, GLS), or tissue characterization parameters (native and post-contrast T1, ECV, T2) between groups (Table 2).

**Table 2:** Results of CMR studies.

	MINOCA *n* = 23	Healthy controls *n* = 26	*p*-value
LV EDV, mL	135.4 ± 39.3	150.8 ± 38.1	0.171
LV EDVi, mL/m^2^	69.4 ± 15.4	76.3 ± 13.8	0.108
LV ESV, mL	48.7 ± 18.1	52.7 ± 18.1	0.445
LV ESVi, mL/m^2^	24.9 ± 7.7	26.6±7.7	0.446
LV SV, mL	86.8 ± 23.0	98.3 ± 22.8	0.086
LV SVi, mL/m^2^	44.7 ± 8.9	49.8 ± 8.0	0.040
LV mass, g	94.7 ± 27.6	96.1 ± 26.5	0.855
LV mass i, g/m^2^	48.6 ± 10.1	48.8±10.7	0.946
LV EF, %	64.8 ± 4.7	65.6 ± 5.4	0.573
LV GLS, %	18.8 ± 2.5	19.1 ± 1.6	0.620
LV GCS, %	21.0 ± 2.0	20.8 ± 2.2	0.655
Native T1, ms	1008 ± 28	1011 ± 22	0.681
Native T2, ms	47 ± 2	46 ± 2	0.891
ECV, %	25.7 ± 2.3	26.2 ± 2.3	0.440

LV, left ventricle; EDV, end-diastolic volume; EDVi, indexed end-diastolic volume; ESV, end-systolic volume; ESVi, indexed end-systolic volume; SV, stroke volume; SVi, indexed stroke volume; EF, ejection fraction; GLS, global longitudinal strain; GCS, global circumferential strain; ECV, extracellular volume fraction.

In contrast, quantitative perfusion revealed significant differences. MINOCA patients demonstrated lower global stress MBF (2.56 ± 0.81 vs. 2.96 ± 0.53 mL/g/min, *p* = 0.044) and lower MPR (1.88 ± 0.59 vs. 2.30 ± 0.55, *p* = 0.013), whereas global rest MBF did not differ between the groups (1.42 ± 0.26 vs. 1.37 ± 0.32 mL/g/min, *p* = 0.539). Perfusion differences were consistent across coronary territories (Table 3). The magnitude of the between-group differences for the primary global perfusion parameters was moderate for stress MBF and moderate-to-large for MPR, as reflected by the standardized effect sizes presented in Table 3 and in Supplementary Table S4.

In multivariable linear regression analyses adjusted for age, sex, and smoking status, MINOCA status remained independently associated with lower global MPR (B = − 0.436, 95% CI − 0.785 to − 0.087, *p* = 0.016). The association between MINOCA status and lower global stress MBF was attenuated after adjustment and did not reach statistical significance (B = − 0.441, 95% CI − 0.903 to 0.021, *p* = 0.061) (Supplementary Tables S1–S2).

**Table 3:** Results of quantitative perfusion analysis.

Perfusion CMR data	MINOCA *n* = 23	Healthy controls *n* = 26	*p*-value	Hedges’ g (95% CI)
Hemodynamic response				
Heart rate at rest, bpm	75 ± 14	74 ± 14	0.745	
Heart rate at stress, bpm	97 ± 13	92 ± 11	0.164	
SBP at rest, mmHg	138 ± 18	132 ± 13	0.156	
SBP at stress, mmHg	138 ± 20	131 ± 14	0.194	
Rest myocardial blood flow (MBF)				
Global	1.42 ± 0.26	1.37 ± 0.32	0.539	0.174 (− 0.380 to 0.726)
LAD-territory	1.48 ± 0.30	1.42 ± 0.37	0.575	
LCX-territory	1.47 ± 0.32	1.45 ± 0.41	0.921	
RCA-territory	1.28 ± 0.25	1.16 ± 0.27	0.112	
Stress myocardial blood flow (MBF)				
Global	2.56 ± 0.81	2.96 ± 0.53	0.044	−0.584 (− 1.145 to − 0.016)
LAD-territory	2.58 ± 0.85	3.08 ± 0.56	0.018	
LCX-territory	2.55 ± 0.82	3.05 ± 0.50	0.013	
RCA-territory	2.39 ± 0.80	2.75 ± 0.57	0.076	
Myocardial perfusion reserve (MPR)				
Global	1.88 ± 0.59	2.30 ± 0.55	0.013	− 0.764 (− 1.333 to − 0.187)
LAD-territory	1.78 ± 0.57	2.28 ± 0.62	0.005	
LCX-territory	1.77 ± 0.57	2.21 ± 0.59	0.012	
RCA-territory	1.92 ± 0.65	2.46 ± 0.60	0.004	

bpm, beats per minute; SBP, systolic blood pressure; DBP, diastolic blood pressure; MBF, myocardial blood flow; MPR, myocardial perfusion reserve; LAD, left anterior descending artery; LCX, left circumflex artery; RCA, right coronary artery. Standardized effect sizes are reported as Hedges’ g with 95% confidence intervals for global perfusion parameters.

Using predefined quantitative CMR thresholds^6^, 10 of 23 MINOCA patients (43%) fulfilled the criteria for findings consistent with coronary microvascular dysfunction, whereas none of the healthy controls fulfilled these criteria.

Interobserver reproducibility of the automated quantitative perfusion analysis was excellent for global stress MBF (ICC 0.993, 95% CI 0.982–0.997) and global MPR (ICC 0.992, 95% CI 0.980–0.997), and good for global rest MBF (ICC 0.858, 95% CI 0.640–0.944) (Supplementary Table S3). Bland–Altman analyses demonstrated close agreement between observers for global stress MBF, global rest MBF, and global MPR without evidence of systematic bias (Supplementary Figures S1–S3).

Finally, 69.6% of MINOCA patients had evidence of non-obstructive atherosclerotic plaque on coronary angiography, despite normal CMR findings.

**Fig. 3:**
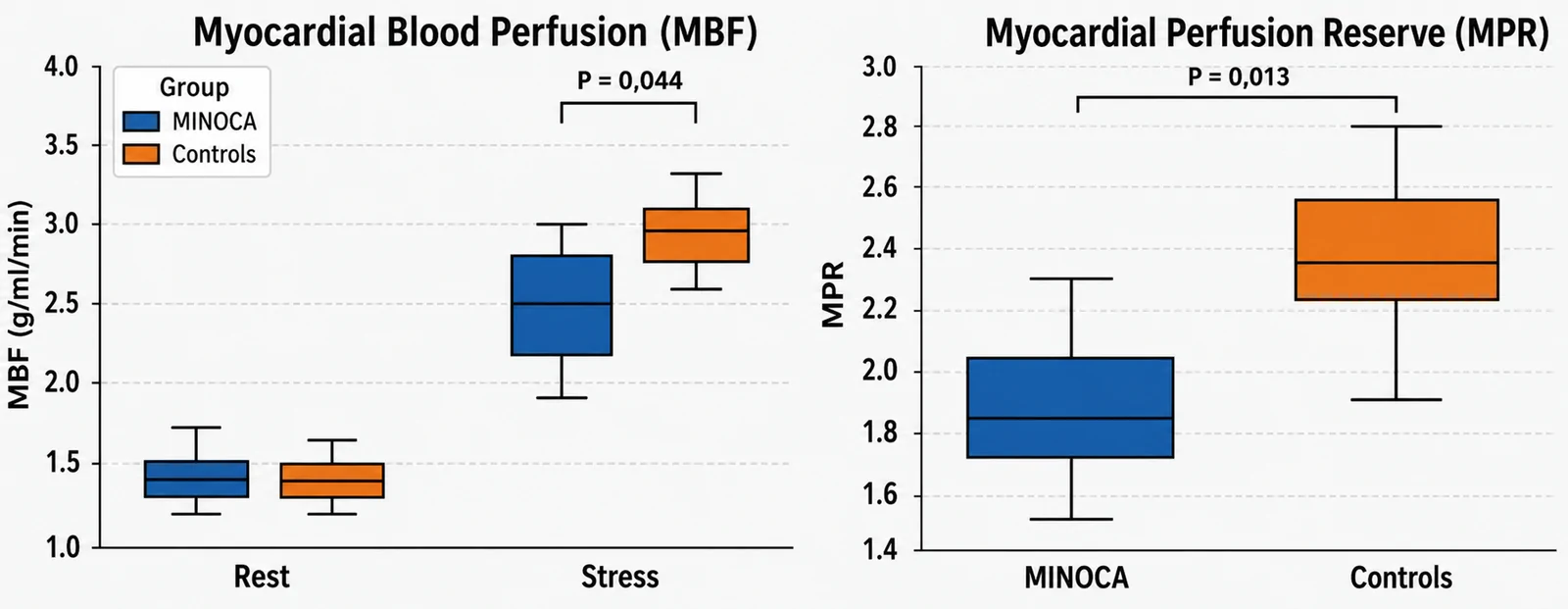
Rest MBF, stress MBF, and MPR in patients with MINOCA compared with healthy controls. MBF myocardial blood flow; MPR, myocardial perfusion reserve.

**Fig. 4:**
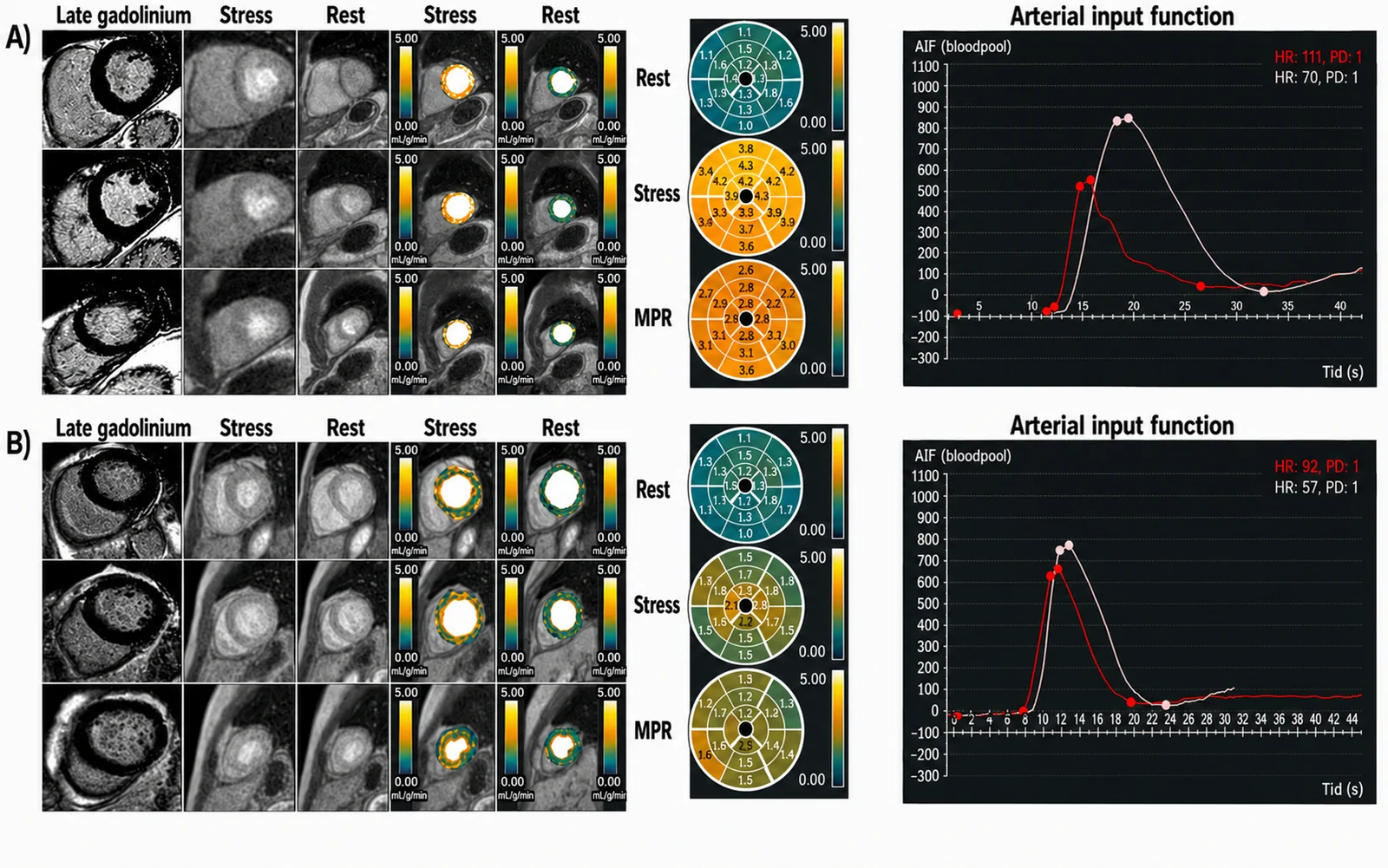
Representative examples of quantitative perfusion maps in stress and rest of a MINOCA patient with and without CMD. (**A**) The first patient shows normal CMR findings and normal perfusion. The polar maps demonstrate normal stress myocardial blood flow (MBF) and normal myocardial perfusion reserve (MPR). (**B**) The second patient shows normal CMR findings and normal coronary angiography, but an abnormal perfusion. The polar maps reveal reduced stress MBF and MPR, while rest perfusion remains normal—findings consistent with coronary microvascular dysfunction (CMD). Both patients showed an appropriate hemodynamic response to regadenoson stress, as illustrated by the heart rate changes and arterial input function curves on the right.

Quantitative perfusion values for rest MBF, stress MBF, and MPR are shown in Fig. 3, and representative perfusion maps are displayed in Fig. 4.

## Discussion

In this prospective study, nearly half of the patients with MINOCA and otherwise normal conventional CMR findings demonstrated reduced stress MBF and impaired MPR on quantitative stress-perfusion CMR, consistent with coronary microvascular dysfunction according to previously proposed CMR thresholds. These findings suggest that impaired coronary microvascular function may contribute to the pathophysiology of a substantial subset of patients with MINOCA whose routine CMR examination is otherwise normal.

Coronary microvascular dysfunction is a biologically plausible mechanism in MINOCA because functional abnormalities of the coronary microcirculation, like limited vasodilatory reserve or increased microvascular resistance, can restrict perfusion during stress despite patent epicardial arteries^20^.

In our cohort, the combination of normal MBF at rest, but reduced stress MBF and MPR, indicates preserved basal perfusion yet an impaired ability to increase flow during demand, which are characteristic features consistent with impaired coronary microvascular function.

Importantly, the observed reduction in myocardial perfusion reserve remained significant after adjustment for age, sex, and smoking status, suggesting that the difference in MPR between MINOCA patients and controls was not fully explained by these measured baseline characteristics.

Our findings are consistent with prior invasive physiology studies. Milzi et al.^14^ showed that angiography-derived index of microvascular resistance (aIMR) was elevated across all three coronary territories in MINOCA patients, regardless of subtype (takotsubo, inflammatory, or “unclear”). Their “unclear” subgroup – characterized by normal CMR findings and a demographic profile similar to our cohort - had significantly higher microvascular resistance than controls (30.6 ± 8.5 vs. 22.1 ± 5.9, *p* < 0.001). Notably, this subgroup represented 45% of all MINOCA patients, which relates to a proportion of normal CMR findings in our cohort. Importantly, Milzi et al.´s data support the hypothesis that CMD may represent a relevant contributor to the pathophysiology of MINOCA.

Additional support comes from long-term imaging studies. In the SMINC-2 cohort, Johansson et al.^13^ reported persistently reduced stress perfusion years after the index MINOCA event, despite normal baseline CMR findings. Their results, obtained using quantitative stress perfusion imaging, are in agreement with our observations and suggest that persistent microvascular abnormalities may occur after MINOCA rather than representing only a transient event-related abnormality.

Our study extends these previous observations by demonstrating that quantitative perfusion abnormalities can already be detected during the early diagnostic evaluation of carefully selected MINOCA patients with otherwise normal conventional CMR findings. Whereas previous quantitative stress-perfusion CMR studies have primarily examined broader MINOCA populations or patients at longer follow-up, our study specifically addresses patients with MINOCA and completely normal conventional CMR findings during the early diagnostic phase. Our findings suggest that quantitative perfusion abnormalities consistent with impaired coronary microvascular function may already be present early after the index event in patients without detectable structural myocardial abnormalities.

In addition, the use of automated quantitative perfusion analysis may facilitate reproducible assessment of myocardial blood flow and support broader clinical implementation of quantitative stress-perfusion CMR. However, further validation across different scanners, populations, and clinical settings is required.

In our cohort, 43% of patients fulfilled previously proposed CMR criteria for findings consistent with CMD^6^. Although the causal relationship between CMD and myocardial injury in MINOCA remains incompletely understood, the clinical significance of CMD is increasingly recognized. CMD has been linked to angina, impaired quality of life, and an elevated risk of major adverse cardiovascular events in patients without obstructive coronary artery disease^21–23^. Detecting perfusion abnormalities consistent with CMD in MINOCA may therefore provide important diagnostic and prognostic information and may inform future therapeutic strategies, even though optimal treatment in this population is not yet defined.

From a clinical perspective, quantitative stress-perfusion CMR may provide incremental diagnostic information in patients with MINOCA whose conventional CMR examination is normal. Identification of perfusion abnormalities consistent with impaired coronary microvascular function may help explain otherwise unexplained myocardial injury and contribute to a more comprehensive diagnostic evaluation. Whether these findings should influence therapeutic decision-making or improve patient outcomes remains to be established in larger prospective multicentre studies. Future studies should determine whether early identification of quantitative perfusion abnormalities can improve risk stratification and guide individualized management strategies in patients with MINOCA.

Notably, more than two-thirds (69.6%) of MINOCA patients with normal CMR demonstrated non-obstructive atherosclerotic plaque on coronary angiography. This observation raises the possibility of an association between non-obstructive atherosclerosis and microvascular dysfunction. Previous studies have reported similar associations^20^, indicating that shared mechanisms such as endothelial dysfunction, inflammation, or diffuse microvascular remodeling may contribute to both processes. Because detailed plaque characterization was not performed, the relationship between plaque burden and quantitative perfusion abnormalities could not be further explored in the present study. The exact nature of this relationship warrants further investigation.

In summary, we found that a substantial proportion of patients with MINOCA and otherwise normal conventional CMR demonstrated impaired quantitative stress perfusion. These perfusion abnormalities were consistent with coronary microvascular dysfunction according to previously proposed CMR thresholds and suggest that impaired coronary microvascular function may be a potential contributor to myocardial injury in a subset of patients with MINOCA. Our findings further support the potential incremental diagnostic value of quantitative stress-perfusion CMR in patients whose routine CMR examination does not reveal an underlying cause.

## Limitations

This study has several limitations. First, the sample size was modest and the study was conducted at a single center, which may limit the generalizability of the findings. In addition, no formal sample size calculation was performed, and the study should therefore be considered exploratory and hypothesis-generating.

Second, although the control group was age- and sex-matched, differences in cardiovascular risk factors and medication use were present between groups. To address potential confounding, multivariable linear regression analyses adjusted for age, sex, and smoking status were performed, demonstrating that the association between MINOCA status and reduced myocardial perfusion reserve remained significant. However, residual confounding from unmeasured factors cannot be excluded.

Third, invasive coronary microvascular assessment was not performed, precluding direct comparison between quantitative CMR-derived perfusion measures and invasive reference methods such as coronary flow reserve or index of microvascular resistance. Therefore, the quantitative perfusion findings should be interpreted as being consistent with, rather than diagnostic of, coronary microvascular dysfunction.

Fourth, multiple quantitative perfusion parameters were compared across global and coronary territory levels without adjustment for multiple comparisons. Although the main analyses were predefined and the findings were biologically consistent across territories, the possibility of type I error cannot be excluded.

Fifth, the absence of longitudinal follow-up prevents assessment of the prognostic implications of the observed perfusion abnormalities consistent with impaired coronary microvascular function and their potential relationship with clinical outcomes.

Finally, although automated perfusion analysis reduces operator dependence, quantitative perfusion measurements may be influenced by technical and physiological factors, including slice selection, motion artifacts, contrast administration, and variability in the hemodynamic response to regadenoson stress. The interobserver reproducibility of the automated analysis was assessed in a subset of participants and demonstrated excellent agreement for stress MBF and MPR, and good agreement for rest MBF (Supplementary Table S3).

Furthermore, although the quantitative perfusion thresholds applied in this study have previously been validated against invasive coronary physiology, they have not been specifically validated for the exact scanner, acquisition protocol, software version, and stress agent used in the present study.

## Conclusions

Early quantitative stress-perfusion CMR identified impaired stress myocardial blood flow and myocardial perfusion reserve in a substantial proportion of patients with MINOCA and otherwise normal conventional CMR findings. These quantitative perfusion abnormalities were consistent with impaired coronary microvascular function in nearly half of the patients, suggesting that coronary microvascular dysfunction may contribute to myocardial injury in a subset of MINOCA patients. Our findings support the potential incremental diagnostic value of quantitative stress-perfusion CMR when routine imaging does not identify an underlying cause, although larger multicentre studies with invasive validation are warranted.

## Data Availability

Data supporting the findings in this study are available from the corresponding author upon reasonable request.
